# Cerebral Venous Sinus Thrombosis in a Patient With Mild Symptoms of COVID-19 Pneumonia

**DOI:** 10.7759/cureus.19885

**Published:** 2021-11-25

**Authors:** Asad Chohan, Farah Chohan, Pedram Rad, George Michel, George Yatzkan

**Affiliations:** 1 Pulmonary Medicine, Corpus Christi Medical Center, Corpus Christi, USA; 2 Internal Medicine, Larkin Community Hospital, South Miami, USA; 3 Pulmonary and Critical Care Medicine, Larkin Community Hospital, South Miami, USA

**Keywords:** thromboembolism, cvst, pneumonia, sars-cov-2, covid-19

## Abstract

Although coronavirus disease 2019 (COVID-19) infection is mainly associated with pneumonia, several non-respiratory complications may also occur. Cerebral venous sinus thrombosis (CVST) is a rare but potentially fatal complication of COVID-19 infection. In order to increase awareness about such life-threatening complications to a large population of patients with otherwise mild COVID-19 infection, we present the clinical course of a 29-year-old unvaccinated female who developed CVST, with eight days of mild COVID-19 infection, that proved fatal despite adequate therapeutic measures. Clinicians should carefully consider the risk of thrombosis in patients who present with COVID-19 infection regardless of the intensity of the disease, including prophylaxis (to reduce the risk of hypercoagulable complications) and treatment beyond discharge. More data and research is needed to identify COVID-19 as an independent risk factor for thromboembolism so that future efforts can be aimed at appropriate management e.g. with prophylactic anticoagulants to avoid such complications. In case of unexplained neurological manifestations in patients with an active or recent COVID-19 infection, early investigations for cerebrovascular integrity should be done by using MRI and magnetic resonance angiography (MRA)/magnetic resonance venography (MRV).

## Introduction

Cerebral venous sinus thrombosis (CVST) is a rare but potentially life-threatening complication of coronavirus disease 2019 (COVID-19) infection. Although COVID-19 infection is mainly associated with pneumonia and respiratory symptoms [1], several non-respiratory complications e.g. cardiovascular [2,3] and thromboembolic [4,5] have also been reported. Different cases of CVST have been reported worldwide in patients with COVID-19 infection, often in patients in the hospital or ICU with progressive inflammation and a prolonged hypercoagulable state. We present a case of a young female, unvaccinated against COVID-19, with no prior risk factors for thromboembolism, who was diagnosed with COVID-19 and initially had mild symptoms and prompt recovery. However, later on, the patient developed CVST which proved fatal.

## Case presentation

A 29 year-old-female presented to the emergency department with progressively worsening somnolence, confusion, and lethargy for the past two days. She was initially diagnosed with COVID pneumonia eight days back, with initial symptoms of mild cough and sore throat and no objective hypoxia and was recommended for home quarantine. The patient became completely symptom-free in two days, followed by neurological symptoms, appearing two days later.

**Figure 1:**
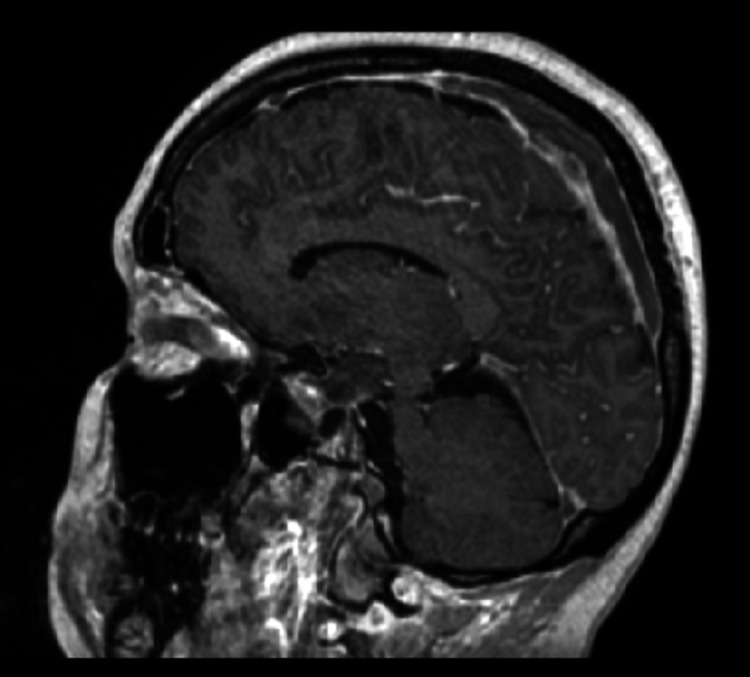
MRI of the brain: acute/early subacute ischemic infarct involving the left frontal, parietal and occipital lobes with effacement of the sulci. No definite hemorrhagic transformation is seen. There is extensive thrombosis involving the dural sinuses.

**Figure 2:**
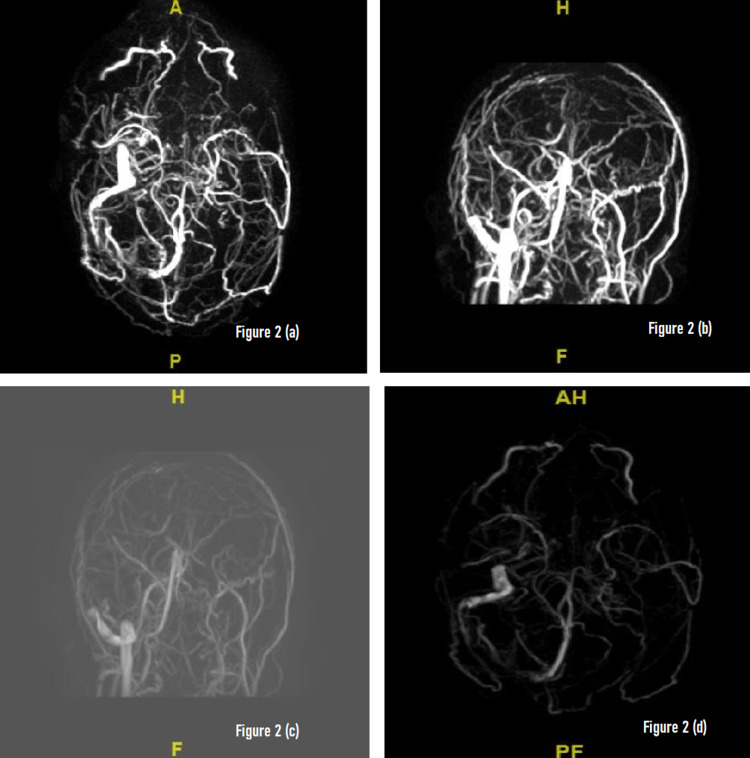
(a-d) Magnetic resonance venography (MRV) of the brain: there is absent flow in the superior sagittal sinus, bilateral transverse sinuses, left sigmoid sinus and within the left jugular bulb highly suspicious for thrombosis. Signal is identified within the distal right sigmoid sinus and right jugular bulb and right internal jugular vein. Normal signal is identified within the internal cerebral veins, basal veins of Rosenthal, vein of Galen and straight sinus.

Patient was reportedly communicative at home before becoming progressively somnolent, with nausea/vomiting, headaches and eventually stopped following directed commands by the time she reached the ED. Her blood pressure was 136/90, heart rate 95, SaO2 99% on room air, respiratory rate 20 and temperature 36.8. Her blood glucose level was 126. Chest X-ray was negative for any acute findings, Non-contrast head CT did not show any acute bleed, fracture or a grossly visible infarct. Rapid antigen COVID-19 test was negative and inflammatory markers were mildly elevated. D-dimer was noted to be >5000. MRI of the brain was significant for acute/early subacute ischemic infarct involving the left frontal, parietal and occipital lobes with effacement of the sulci. Extensive thrombosis involving the dural sinuses was seen (Figure 1). MRV revealed absent flow within superior sagittal sinus, bilateral transverse sinuses, sigmoid sinus and left jugular bulb compatible with extensive cavernous venous thrombosis (Figure 2).

The patient was subsequently started on heparin drip and neurology and neurosurgery teams were brought on board. Cerebral arteriogram was done and the patient was sedated/intubated and transferred to the ICU. Cerebral venous sinus thrombectomy was performed with clot aspiration and thrombolytic therapy. Significant clot removal and partial flow restoration was achieved but a severe occlusive clot remained. Unfortunately, the patient continued to show further neurological deterioration to the point where she lost all apparent brainstem reflexes and withdrawal responses. This was reflective of worsening cerebral edema and increased intracranial pressure as a result of venous obstructive disease. It was deemed an unsurvivable event and no further neurosurgical intervention seemed appropriate. After a repeat EEG showing no significant brain activity and a negative brain flow study, the patient was legally determined to be brain-dead. Being an organ donor, appropriate protocols were followed with the family on board.

## Discussion

The emergence of severe acute respiratory syndrome coronavirus 2 (SARS-CoV-2) in late 2019 led to a global pandemic healthcare crisis across the world resulting in more than 247 million cases worldwide and 5 million deaths to date. Although COVID-19 primarily manifests as a lung infection, with symptoms ranging from those of a mild upper respiratory infection to severe pneumonia and acute respiratory distress syndrome, other multisystemic manifestations of this disease and related complications are becoming more commonly recognized [6].

The global pandemic of COVID-19 has presented with multiple forms, often linked to a hypercoagulable state resulting in venous thrombotic events [7]. Our case of a young female with mild symptoms of COVID-19 and appropriate recovery and later on sudden development of a CVST without any other known risk factors sheds light on this potentially lethal complication in all COVID-19 patients. It has to be noted that the hypercoagulability related to COVID-19 infection and the use of prophylactic anticoagulants has been a subject of debate over the past 1.5 years. Moreover, the suspicion of new variants with different intensity of inflammation and hypercoagulability has been another cause of academic curiosity. CVST is a fairly uncommon disease, with an annual incidence ranging between 1.32 and 1.57 per 100 000, [8,9] being more prevalent in younger females than men [10]. CVST often presents as one of the three syndromes: isolated intracranial hypertension, encephalopathy, and focal syndrome. CVST can cause stroke due to focal cerebral infarction and haemorrhage which can be identified on brain imaging in 46.5% and 39.3% of presentations, respectively [11]. Diagnosis is usually done by urgent neuroimaging with cranial CT and CT venography or MRV. Anticoagulation with heparin is usually the initial treatment of choice even in the presence of intracerebral haemorrhage, and has reportedly resulted in a good outcome [12].

Over the past few months, multiple cases of CVST have been reported but the usual patient population has been patients with COVID-19 with moderate to severe inflammation and overt symptoms. Generally, those cases have been generally managed in the hospital or in the ICU and thromboembolic phenomena have been reported only after a prolonged disease. It was commonly believed by clinicians that COVID-19 in patients with no major comorbidities, young age and active lifestyle, and adequate mobility, have minimal complications if their COVID-19 infection recovers quickly. In a case report by Abouhashem et al., two young patients with no risk factors for hypercoagulable state besides a prior COVID infection are discussed. Both patients died within one week of their initial symptoms despite aggressive and prompt medical and surgical management, hence pointing towards a possible association between COVID-19 and CVST [13]. Another case report by Bolaji et al. discussed a patient who developed extensive CVST with bilateral venous cortical infarcts and acute cortical haemorrhage following COVID-19 infection [14].

## Conclusions

Our case provides an insight into the possible risks of devastating complications in a large population of patients who experience only mild symptoms of COVID-19 infection. This case can also serve as a guide to further clinical investigation on the hypercoagulability associated with the infection of COVID-19 regardless of the clinical progression of the disease or the clinical signs of inflammation. Future research should be aimed at investigating COVID-19 as an independent risk factor for thromboembolism. Once it is determined that the hypercoagulability of COVID-19 is clinically significant regardless of the intensity of the disease, future efforts can be aimed at management with prophylactic anticoagulants to reduce the risk of possibly fatal complications. In case of unexplained neurological manifestations in patients with an active or recent COVID-19 infection, early investigations for cerebrovascular integrity should be done by using magnetic resonance angiography (MRA)/MRV. 
